# Oral delivery of exenatide-loaded hybrid zein nanoparticles for stable blood glucose control and β-cell repair of type 2 diabetes mice

**DOI:** 10.1186/s12951-020-00619-0

**Published:** 2020-04-28

**Authors:** Xiaoyan Bao, Kang Qian, Ping Yao

**Affiliations:** 1 State Key Laboratory of Molecular Engineering of Polymers, Collaborative Innovation Center of Polymers and Polymer Composite Materials, Department of Macromolecular Science, Fudan University, Shanghai, 200438 China; 2 Key Laboratory of Smart Drug Delivery, Ministry of Education, School of Pharmacy, Fudan University, Shanghai, 201203 China

**Keywords:** Diabetes, Exenatide, Nanoparticle, Absorption enhancer, Oral delivery, Zein

## Abstract

**Background:**

Exenatide is an insulinotropic peptide drug for type 2 diabetes treatment with low risk of hypoglycemia, and is administrated by subcutaneous injection. Oral administration is the most preferred route for lifelong treatment of diabetes, but oral delivery of peptide drug remains a significant challenge due to the absorption obstacles in gastrointestinal tract. We aimed to produce exenatide-loaded nanoparticles containing absorption enhancer, protectant and stabilizer using FDA approved inactive ingredients and easy to scale-up method, and to evaluate their long-term oral therapeutic effect in type 2 diabetes db/db mice.

**Results:**

Two types of nanoparticles, named COM NPs and DIS NPs, were fabricated using anti-solvent precipitation method. In COM NPs, the exenatide was complexed with cholic acid and phosphatidylcholine to increase the exenatide loading efficiency. In both nanoparticles, zein acted as the cement and the other ingredients were embedded in zein nanoparticles by hydrophobic interaction. Casein acted as the stabilizer. The nanoparticles had excellent lyophilization, storage and re-dispersion stability. Hypromellose phthalate protected the loaded exenatide from degradation in simulated gastric fluid. Cholic acid promoted the intestinal absorption of the loaded exenatide via bile acid transporters. The exenatide loading efficiencies of COM NPs and DIS NPs were 79.7% and 53.6%, respectively. The exenatide oral pharmacological availability of COM NPs was 18.6% and DIS NPs was 13.1%. COM NPs controlled the blood glucose level of the db/db mice well and the HbA_1c_ concentration significantly decreased to 6.8% during and after 7 weeks of once daily oral administration consecutively. Both DIS NPs and COM NPs oral groups substantially increased the insulin secretion by more than 60% and promoted the β-cell proliferation by more than 120% after the 7-week administration.

**Conclusions:**

Both COM NPs and DIS NPs are promising systems for oral delivery of exenatide, and COM NPs are better in blood glucose level control than DIS NPs. Using prolamin to produce multifunctional nanoparticles for oral delivery of peptide drug by hydrophobic interaction is a simple and effective strategy.

## Background

The number of diabetics has grown to 464 million, among which type 2 diabetes is the most common type, and the deaths caused by diabetes and the complications have reached to 4.2 million in 2019, according to the estimation of International Diabetes Federation [1]. Blood glucose control, which is the primary approach to reduce diabetes complications and mortality, remains a challenge in diabetes treatment [2–4]. Glucagon-like peptide-1 (GLP-1]) is a gut released incretin hormone responding to nutrient ingestion, and is a potent glucose-dependent insulinotropic peptide with low risk of hypoglycemia [5]. Exenatide (EXE), which consists of 39 amino acid residues, is the first marketed GLP-1 analog for type 2 diabetes treatment and is administrated by subcutaneous injection [6, 7]. Considering the lifelong treatment, peroral administration of EXE or other GLP-1 analogs is the most preferred route for diabetics that can avoid injection pain and site infection [8, 9]. In addition, oral delivery of GLP-1 analogs is more similar to the naturally secreted GLP-1, and can reduce the systemic exposure and attendant side-effects [10]. However, the oral bioavailability of peptide drug is extremely low because of the absorption obstacles in gastrointestinal tract, that is, large pH gradient, proteases, mucus and enterocyte barriers [11–13]. Various nano-sized particles, liposomes, micelles and emulsions have been fabricated to protect the encapsulated peptide drug and enhance the absorption in gastrointestinal tract [14–18]. By now, although great efforts have been made in developing oral delivery systems of peptide drug, the applications are very limited. The challenge is not only to have desired oral therapeutic efficacy but also have approved biocompatibility and safety as well as feasibility of industrial production [19–21].

Transcellular and paracellular transports are the main absorption routes of orally administrated peptide drug in intestinal tract. For example, we previously reported insulin-loaded nanoparticles produced by cholic acid modified chitosan derivative and hypromellose phthalate, and demonstrated that the nanoparticles were an effective oral delivery system of insulin via bile acid transporters and reversibly opened tight junctions [22]. However, the opening of the tight junctions may increase the risk of diarrhea, edema and autoimmune diseases [23, 24]. Given the lifelong treatment of diabetes, it may be safer to keep the tight junctions unopened for oral delivery of peptide drug.

In this study, we aimed to utilize bile acid transporters and other transcellular pathways to deliver peptide drug orally, and aimed to produce peptide drug-loaded nanoparticles containing absorption enhancer, protectant and stabilizer using FDA approved inactive ingredients and easy to scale-up method. We used zein as cement to embed EXE, phospholipid, cholic acid, hypromellose phthalate and casein in nano-sized aggregates. Zein is one of prolamins [25]. Because of the high molar percentage of hydrophobic amino acid residues, zein is soluble in 60–95% ethanol solution but insoluble in water [26]. The drug and nutriment that can dissolve in the ethanol solution can be encapsulated in zein nanoparticles by anti-solvent precipitation method [27, 28]. Recently, Ji et al. reported insulin-loaded zein-carboxymethylated short-chain amylose nanocomposites with chitosan coating [29]. Phospholipids are the major components of cell membranes. The complexation of phospholipid with peptide can improve in vivo stability of the peptide [30] and promote the transportation across intestinal epithelium [31, 32]. Bile acid and its derivatives facilitate the absorption of lipophilic nutrients [33]. Macromolecules and nanoparticles conjugated with bile acid or its derivative covalently can be internalized via apical sodium-dependent bile acid transporter (ASBT) at the distal small intestine, [34] and can be transported to the basolateral side via cytosolic ileal bile acid-binding protein (IBABP) [35, 36] Hypromellose phthalate (HP), an enteric material, can protect the encapsulated peptide in stomach [37]. Casein is able to stabilize zein nanoparticles by suppressing their hydrophobic aggregation in aqueous solution [38]. We chose cholic acid (CA), phosphatidylcholine (PC), HP and casein as the absorption enhancers, protectants and stabilizers, and EXE as the peptide drug to fabricate hybrid zein nanoparticles.

Two types of nanoparticles, named DIS NPs and COM NPs, were produced by antisolvent method. In DIS NPs, EXE, PC, CA, HP and casein were directly dispersed and embedded in zein nanoparticles by hydrophobic interaction. In COM NPs, EXE was complexed with PC and CA to increase the EXE loading efficiency. We hypothesized that the absorption enhancers dispersed in the interiors of the nanoparticles would be exposed to the surfaces when the nanoparticles were gradually eroded by proteases in gastrointestinal tract, and the exposed absorption enhancers could promote the intestinal absorption of the encapsulated EXE. To prove our hypothesis, the intestinal absorption mechanism, oral pharmacokinetics and pharmacodynamics of the nanoparticles were investigated, and the long-term oral hypoglycemic efficacy and β-cell repair effect in type 2 diabetes db/db mice were evaluated.

## Materials and methods

### Materials

Exenatide (EXE) was purchased from Chinese Peptide Company Co., Ltd. (Hangzhou, Zhejiang, China). Zein, casein, cholic acid (CA, 98%) and glycocholic acid hydrate (GCA, ≥ 97%) were from Sigma-Aldrich (Shanghai, China). Hypromellose phthalate (HP, HP-55S) was from Huzhou Mizuda Hope Bioscience Co., Ltd. (Huzhou, Zhejiang, China), and was purified by dialysis and lyophilization. Phosphatidylcholine (PC, egg yolk lecithin PL-100 M, 80% phosphatidylcholine) was from A.V.T. Pharmaceutical Co., Ltd. (Shanghai, China). Deoxycholic acid (DCA, > 99%), rhodamine B isothiocyanate (RITC, conjugation grade), pepsin (from porcine stomach, 3000–3500 NFU/mg), pancreatin (Pancreatin-8.0, from porcine pancreas, protease 214 USP.U/mg, amylase 214 USP.U/mg and lipase 24.2 USP.U/mg), colchicine (reagent grade) and chlorpromazine (> 98%) were from Sangon Biotech Co., Ltd. (Shanghai, China). Sulfo-cyanine 5 NHS ester (Cy5, analytical grade) was from Lumiprobe Corporation (Maryland, USA). Fluorescein isothiocyanate (FITC) was from Tokyo Chemical Industry Co. Ltd. (Tokyo, Japan). All culture reagents and materials were from Thermo Fisher Scientific Inc. (Shanghai, China). All other reagents were from Sinopharm Chemical Reagent Co., Ltd. (Shanghai, China).

### Preparation of EXE, PC and CA complex micelles (EXE/PC/CA Complex)

A solution was prepared by mixing 1 mL EXE methanol solution with 1.6 mg/mL EXE, 1 mL CA methanol solution with 5 mg/mL CA, and 2 mL PC dichloromethane solution with 10 mg/mL PC together. The organic solvent of the mixed solution was removed by rotary evaporation followed by nitrogen flow. EXE/PC/CA Complex solution was obtained after the dried mixture was re-dispersed in 2 mL CA ethanol solution with 2.5 mg/mL CA.

### Preparation of hybrid zein nanoparticles containing EXE/PC/CA Complex, HP and casein (COM NPs)

Casein was dissolved in deionized water with 2.5 mg/mL casein, and the solution was adjusted to pH 7.4. An ethanol and water mixed solution was prepared by mixing 2 mL of the EXE/PC/CA Complex solution described above, 1 mL HP ethanol/water (4/1, v/v) solution with 20 mg/mL HP, and 1 mL zein ethanol/water (9/1, v/v) solution with 100 mg/mL zein together. Subsequently, 16 mL of the casein aqueous solution was poured into the ethanol and water mixed solution, and the solution was stirred at room temperature for 3 h to evaporate the ethanol. After supplementing deionized water in the mixed solution to the final volume of 20 mL, COM NPs solution was obtained.

### Preparation of hybrid zein nanoparticles containing EXE, PC, CA, HP and casein (DIS NPs)

HP, zein and casein solutions were prepared as described above. EXE was dissolved in deionized water with 3.2 mg/mL concentration; PC was dissolved in ethanol with 20 mg/mL concentration; CA was dissolved in deionized water with 20 mg/mL concentration and final pH 7.4. The EXE solution of 0.5 mL was mixed with 0.5 mL CA solution, then, 1 mL PC solution, 1 mL HP solution and 1 mL zein solution were added one by one, followed by addition of 16 mL casein solution. The mixed solution was stirred at room temperature for 3 h to evaporate the ethanol. After supplementing deionized water in the mixed solution to the final volume of 20 mL, DIS NPs solution was obtained.

In addition, the nanoparticle solutions with different compositions and concentrations, which are listed in Tables 1, 2 as well as Additional file 1: Table S1, were produced using the same method.

**Table 1:** Constituents and properties of EXE-loaded zein NPs without EXE/PC/CA Complex (n = 3)

Formulation	EXE	Zein	PC	CA	HP	Casein	D_h_ (nm)	PDI	ζ-Potential (mV)	LE (%)
	mg/mL									
DIS NPs	0.08	5	1	0.5	1	2	177 ± 1	0.06 ± 0.04	− 15.1 ± 1.4	53.6 ± 0.3
DIS-1 NPs	0.08	3.75	1	0.5	1	2	187 ± 2	0.16 ± 0.02	− 32.3 ± 1.7	44.9 ± 1.8
DIS-2 NPs	0.08	2.5	1	0.5	1	2	168 ± 1	0.13 ± 0.03	− 38.4 ± 1.9	31.1 ± 1.9

**Table 2:** Constituents and properties of EXE/PC/CA Complex-loaded zein NPs (n = 3)

Formulation	EXE	Zein	PC	CA	HP	Casein	D_h_ (nm)	PDI	ζ-Potential (mV)	LE (%)
	mg/mL									
EXE/PC/CA	0.08	0	1	0.5^a^	0	0	141 ± 1	0.27 ± 0.02	− 2.3 ± 0.5	82.7 ± 0.4
COM NPs	0.08	5	1	0.5^a^	1	2	241 ± 3	0.07 ± 0.01	− 13.2 ± 1.1	79.7 ± 0.1
COM-1 NPs	0.08	5	1	0.5^a^	1	0	256 ± 4	0.02 ± 0.01	–	83.7 ± 0.1
COM-2 NPs	0.08	5	1	0.5^a^	1	4	170 ± 1	0.12 ± 0.04	–	79.1 ± 0.3
COM-3 NPs	0.08	3.75	1	0.5^a^	1	2	220 ± 1	0.04 ± 0.04	–	70.5 ± 0.5
COM-4 NPs	0.08	2.5	1	0.5^a^	1	2	141 ± 2	0.13 ± 0.02	–	68.2 ± 0.8
COM-5 NPs	0.08	5	1	0	1	2	193 ± 1	0.15 ± 0.02	–	77.9 ± 0.2
COM-6 NPs	0.08	5	1	0.5^a^	0	2	421 ± 9	0.22 ± 0.04	–	73.0 ± 0.4
COM-7 NPs	0.08	5	1	0.5^b^	1	2	216 ± 1	0.08 ± 0.02	–	75.4 ± 0.6
COM-8 NPs	0.08	5	1	0.5^c^	1	2	258 ± 5	0.07 ± 0.04	–	82.1 ± 0.1

^a^The CA was cholic acid

^b^The CA was deoxycholic acid

^c^The CA was glycocholic acid

### Characterization of the NPs

FITC conjugated EXE (FITC-EXE) was synthesized [39]. FITC-EXE was used to prepare FITC-EXE/PC/CA Complex, FITC-COM NPs and FITC-DIS NPs. The free FITC-EXE in the NPs solution was separated by ultrafiltration (cutoff molecular weight 100 kDa, MicroconYM-100, Millipore) and measured using a fluorescence microplate reader (Cytation3, BioTek). The EXE loading efficiency (LE) of the NPs solution was calculated using the equation:

$$ {\text{LE }}\;\left( {{\text{\% , w/w}}} \right)\;{ = }\;\frac{{{\text{Total FITC-EXE }}\; - \;{\text{Free FITC-}}\,{\text{EXE}}}}{{{\text{Total FITC-}}{\text{EXE }}}}  \;{{ \times }}\; 1 0 0 {\text{\% }} $$

Z-Average hydrodynamic diameter (D_h_), polydispersity index (PDI) and ζ-potential of the NPs solution were measured at 25 ℃ on a dynamic light scattering (DLS) instrument (Zetasizer Nano ZS90, Malvern). Morphology of the NPs was observed on a transmission electron microscope (CM120, Philips) and field emission scanning electron microscope (Ultra 55, Zeiss).

### In vitro permeability across excised small intestinal walls of mice

Healthy male ICR mice (20  ± 2 g) were from Sino-British SIPPR/BK Lab Animal Ltd. (Shanghai, China). In vitro transports of free EXE and the NPs across excised duodenum, jejunum and ileum walls of mice were investigated as reported in the literature [40]. Free FITC-EXE, FITC-DIS NPs or FITC-COM NPs solution of 0.1 mL with EXE concentration of 80 μg/mL was injected into the duodenum, jejunum or ileum segment after the both ends were tied. The intestinal segment was immersed in 3 mL Krebs–Ringer solution and incubated at 37 °C. The transported FITC-EXE in the solution was analyzed on the fluorescence microplate reader. The apparent EXE permeability coefficient (P_app_) was calculated using the equation:

$$ P_{app} \, = \,\frac{Q}{{AC_{0} t}} $$

where *Q* was the cumulative amount (ng) of the transported FITC-EXE across the intestine wall, *A* was the diffusion area (cm^2^) of the intestinal segment, *C*_*0*_ was the initial FITC-EXE concentration (ng/mL), and *t* was the duration time (s) of the experiment.

### Ex vivo fluorescence imaging of intestinal sections

RITC-labelled zein (RITC-Zein) and Cy5-labelled EXE (Cy5-EXE) were separately prepared as described in the literature [22]. RITC and Cy5-labelled COM NPs and DIS NPs were prepared using RITC-Zein and Cy5-EXE. After fasting for 12 h with free access to water, the healthy mice were orally administrated with Cy5-EXE and the double-labelled NPs separately at 2.4 mg/kg EXE dose, which was fourfold of the once daily dose in the hypoglycemic experiments to increase the fluorescence intensities. The mice were sacrificed at 2 h post-administration, and the duodenum, jejunum and ileum segments were taken out, opened and gently washed with saline. The cryostat sections of the segments were stained with DAPI fluoromount-G^TM^, sealed and then observed on a confocal laser scanning microscope (CLSM, C2^+^, Nikon).

### EXE oral bioavailability evaluation

After fasting for 12 h with free access to water, the healthy mice were separately administrated with free EXE solution via subcutaneous injection (s.c.) at 0.06 mg/kg EXE dose as well as with DIS NPs and COM NPs solutions per os (p.o.) at 0.6 mg/kg EXE dose. After administration, the mice had free access to standard chow and water. At predetermined time points, blood sample was collected from the eye ground vein. The EXE concentration in the plasma was analyzed using EXE Elisa kit (Phoenix Pharmaceuticals Inc., Burlingame, California, USA). Relative EXE oral bioavailability (BA) versus subcutaneous injection was calculated according to the area under the EXE plasma concentration–time curve during 0–24 h (*AUC*_0–24_) using the following equation:

$$ {\text{BA(\%)}} = \,\frac{{\left( {AUC_{0 - 24 } } \right)_{p.o.} \; \times \;Dose_{s.c.} }}{{\left( {AUC_{0 - 24} } \right)_{s.c.} \; \times \;Dose_{p.o.} }}\; \times \;100\% .$$

### Oral pharmacodynamics evaluation

Male db/db mice from Model Animal Research Center of Nanjing University (Nanjing, Jiangsu, China) with blood glucose above 19.4 mM (21.5 ± 1.7 mM) were randomly divided into 5 groups (n = 6 per group). Free EXE solution was injected subcutaneously at 0.06 mg/kg dose. DIS NPs and COM NPs were orally administered at 0.6 mg/kg EXE dose. Saline was orally administered as the control. At predetermined time points, the blood sample from the tail vein was collected and the blood glucose level (BGL) was measured using a glucometer (ACCUCHEK Active, Roche). The mice had free access to water and standard chow during the whole experiment. EXE oral pharmacological availability (PA) was calculated according to the area above the relative BGL-time curve (*AAC*) using the following equation:

$$ PA \left( \% \right)\; = \;\frac{{(AAC_{NPs,  p.o.} \, - \,AAC_{Saline,p.o.} )/Dose_{NPs,p.o.} }}{{(AAC_{EXE,  s.c.} \, - \,AAC_{Saline,p.o.} )/Dose_{EXE,s.c.} }}\; \times \;100\%. $$

### Hypoglycemic effect evaluation and related physiological index analysis during and after 7-week administration

For 7-week experiment, the db/db mice with 23.7 ± 2.5 mM blood glucose were chosen, and the once daily EXE doses were the same as those described above. The BGL and body weight were measured before administration at predetermined time points. The mice had free access to water and standard chow during the whole experiment.

After the 7-week administration, the db/db mice were fasting for 12 h, and then the blood sample was collected from the eye ground vein. The HbA_1c_ concentration in the whole blood sample was analyzed using HbA_1c_ assay kit (Crystal Chem Inc., Elk Grove Village, Illinois, USA). The insulin and C-peptide concentrations in the plasma of the sample were analyzed using the corresponding Elisa kit (Crystal Chem Inc.).

### Insulin immunostaining of pancreas

After the 7-week administration and blood sampling, the db/db mice were immediately sacrificed, and the pancreatic tissues were taken out. After fixation in 4% paraformaldehyde solution, each of the pancreatic tissues was embedded in paraffin, and then sectioned across the largest transverse section of the pancreas [41]. The insulin-immunostained sections were prepared as reported, [42] and were observed on a fluorescence microscope (BX53, OLYMPUS). The β-cell areas of the images were measured using Image J software.

### Statistical analysis

All the experiment data were shown as mean ± standard deviation. Data analysis was performed using two-sample t-Test (Origin pro 8.5 software). The difference was significant at P < 0.05.

## Results

### Preparation and characterization of the nanoparticles

**Fig. 1:**
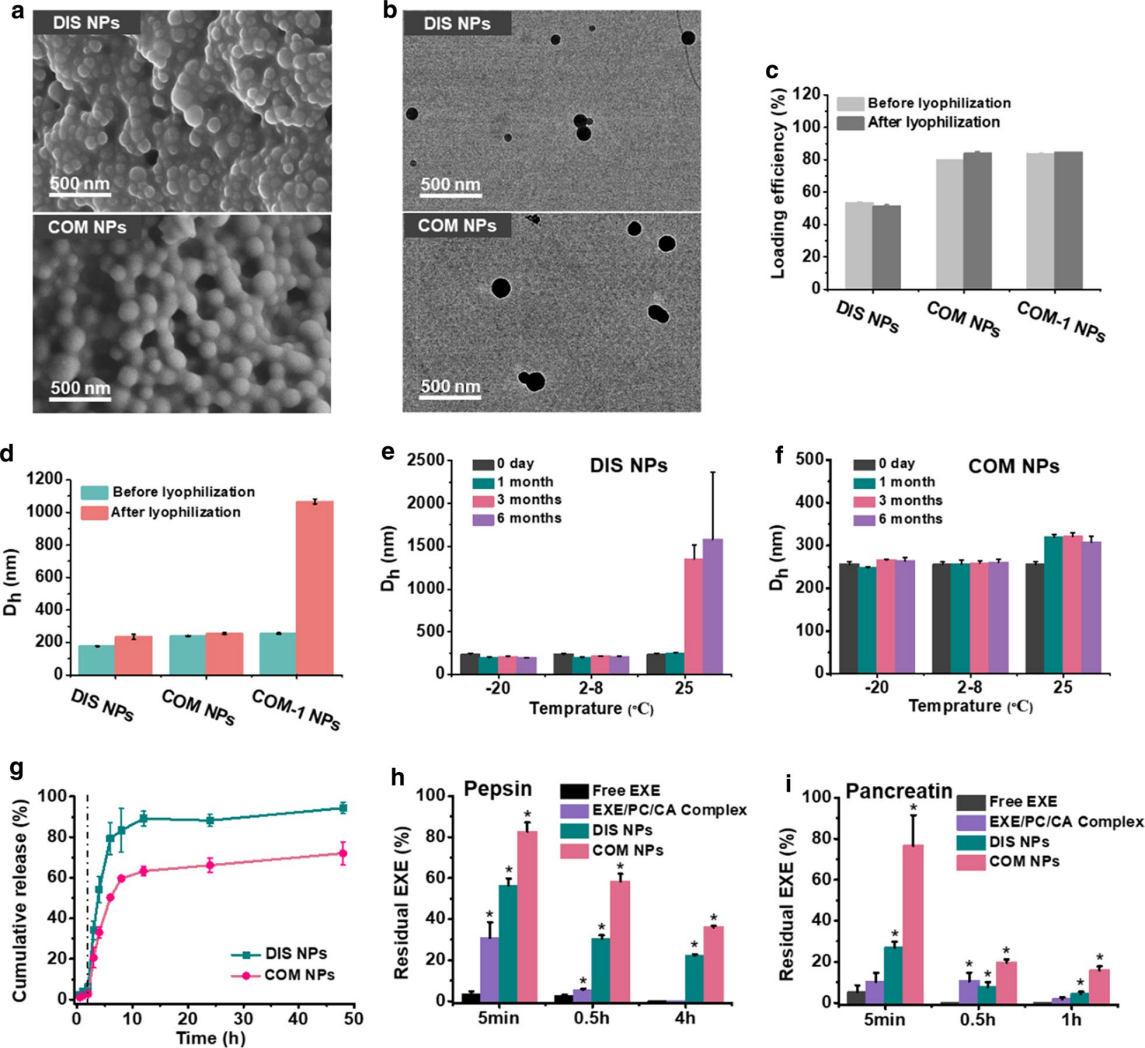
**a** FESEM and **b** TEM images of DIS NPs and COM NPs. **c** Loading efficiencies and **d** D_h_ values of DIS NPs, COM NPs and COM-1 NPs (without casein) before and after lyophilization then re-dispersion; D_h_ values of the re-dispersed **e** DIS NPs and **f** COM NPs after lyophilization and storage at − 20, 2–8 and 25 °C for different periods. **g***In vitro* EXE releases from DIS NPs and COM NPs in pH 2.0 HCl solution (0–2 h) and pH 7.4 PBS solution (2–48 h). Residual EXE of free EXE, EXE/PC/CA Complex, DIS NPs and COM NPs samples after incubations in **h** pepsin solution at pH 2.0 and **i** pancreatin solution at pH 7.4, *P < 0.05 compared with free EXE. (n = 3)

Two types of nanoparticles were produced by antisolvent method. For the preparation of DIS NPs, the casein aqueous solution was mixed with the ethanol solution containing EXE, CA, PC, HP and zein. After the mixing, the sharply reduced ethanol concentration resulted in the formation of zein aggregates, and the other ingredients were dispersed and embedded in the zein aggregates through hydrophobic interaction. Decreasing the zein concentration from 5 to 3.75 and 2.5 mg/mL in the formulations of DIS NPs, DIS-1 NPs and DIS-2 NPs (Table 1), the EXE loading efficiency decreased from 53.6% to 44.9% and 31.1%. These results demonstrated that the hydrophobic aggregation of the zein resulted in the EXE loading in the nanoparticles. Because of the hydrophilicity, the EXE tended to diffuse into the solution. The EXE loading efficiency of the optimized formulation, DIS NPs, was only 53.6%. To increase the hydrophobicity of EXE and thus the EXE loading efficiency, EXE/PC/CA complex micelles were produced firstly, and then the complex micelles as well as HP and casein were loaded in zein nanoparticles. The nanoparticles containing the complex micelles with different compositions and concentrations were produced as shown in Table 2. COM NPs, the optimized formulation containing the complex micelles, had EXE loading efficiency of 79.7%, significantly higher than DIS NPs. COM NPs and DIS NPs had D_h_ values of 241 and 177 nm with narrow size distributions of 0.07 and 0.06, and their ζ-potentials were − 13.2 and − 15.1 mV, respectively. The FESEM (field emission scanning electron microscopy) and TEM (transmission electron microscopy) images (Fig. 1a, b) show that COM NPs and DIS NPs are spherical nanoparticles with homogenous internal structure.

### Lyophilization, storage and re-dispersion stability

After lyophilization without additional cryoprotectant and then re-dispersion in deionized water, the loading efficiencies of DIS NPs and COM NPs did not change significantly as shown in Fig. 1c. DIS NPs and COM NPs only increased their D_h_ values about 58 and 15 nm (Fig. 1d), respectively, indicating that both DIS NPs and COM NPs were well re-dispersible after lyophilization. COM-1 NPs, which had the same formulation as COM NPs but no casein (Table 2), aggregated heavily after lyophilization and re-dispersion (Fig. 1d). The difference between COM NPs and COM-1 NPs demonstrated that casein acted as the cryoprotectant for DIS NPs and COM NPs. Furthermore, the lyophilized DIS NPs and COM NPs did not aggregate significantly after 6 months of storage at − 20  and 2–8 ℃ as shown in Fig. 1e, f. This excellent lyophilization, storage and re-dispersion stability of DIS NPs and COM NPs will be of great value for their application.

### In vitro release of the loaded EXE

In vitro releases of EXE from DIS NPs and COM NPs were investigated. At the first 2 h, the release medium was pH 2.0 HCl solution, in which DIS NPs and COM NPs only released about 6% of the total EXE (Fig. 1g). The photos in Additional file 1: Fig. S1 show that both DIS NPs and COM NPs precipitated in pH 2.0 solution due to the protonation of the carboxyl groups of HP and CA at low pH [22, 43]. Possibly, the unloaded EXE molecules were adsorbed and/or involved in the precipitates that limited their diffusion. The EXE release behavior in pH 2.0 solution suggests that DIS NPs and COM NPs can protect the EXE from digestion in stomach. When the release medium was changed to pH 7.4 PBS, the precipitates disappeared immediately. During the first 2 h in PBS, about 50% and 30% of the EXE were respectively released from DIS NPs and COM NPs samples, which corresponded to the fast diffusion of the free EXE molecules. After 48 h release, about 7% and 28% of the EXE were respectively retained in the DIS NPs and COM NPs, indicating that COM NPs have better sustained release behavior than DIS NPs.

### Protection of the loaded EXE from degradation

Zein has a low digestibility which can protect the encapsulated contents in gastrointestinal tract, [44, 45] and casein as the stabilizer does not significantly alter the digestion of zein [46]. In this study, much higher weight ratios of pepsin:zein and pancreatin:zein were used than the ratios reported in the literature [46] to accelerate the digestion of zein and better evaluate the difference in EXE protection between DIS NPs and COM NPs. For free EXE samples, only 4% of the EXE was undegraded after 5 min of the incubation in pH 2.0 pepsin solution as shown in Fig. 1h. For DIS NPs and COM NPs samples, 22% and 36% of the EXE remained undegraded, respectively, even after 4 h of the incubation, indicating that DIS NPs and COM NPs can effectively protect the loaded EXE from digestion in stomach. In pH 7.4 pancreatin solution, the EXE degradation rate of COM NPs was much slower than the rate of DIS NPs (Fig. 1i). This result can be explained by the fact that COM NPs had higher EXE loading efficiency, and indicates that COM NPs have better EXE protection effect against protease digestion than DIS NPs.

### In vitro and in vivo biocompatibility

Additional file 1: Fig. S2 shows that the Caco-2 cell viabilities were above 90% after 48 h incubations with the culture media containing 60–958 μg/mL DIS NPs and COM NPs. To further prove the good biocompatibility of the NPs, in vivo biocompatibility of the NPs was evaluated after 15 days of consecutive oral administration at the NPs dose of 359 mg/kg once daily, which was equal to 3 mg/kg EXE dose and was fivefold of the once daily dose in the hypoglycemic experiments. The hematoxylin–eosin stained histological images of the mouse organ sections of the two NPs groups did not show distinct change compared with the images of the saline group after the consecutive oral administration (Additional file 1: Fig. S3). The results of the cytotoxicity and histological analysis confirm that both DIS NPs and COM NPs are biocompatible.

### Permeability across Caco-2 cell monolayers

**Fig. 2:**
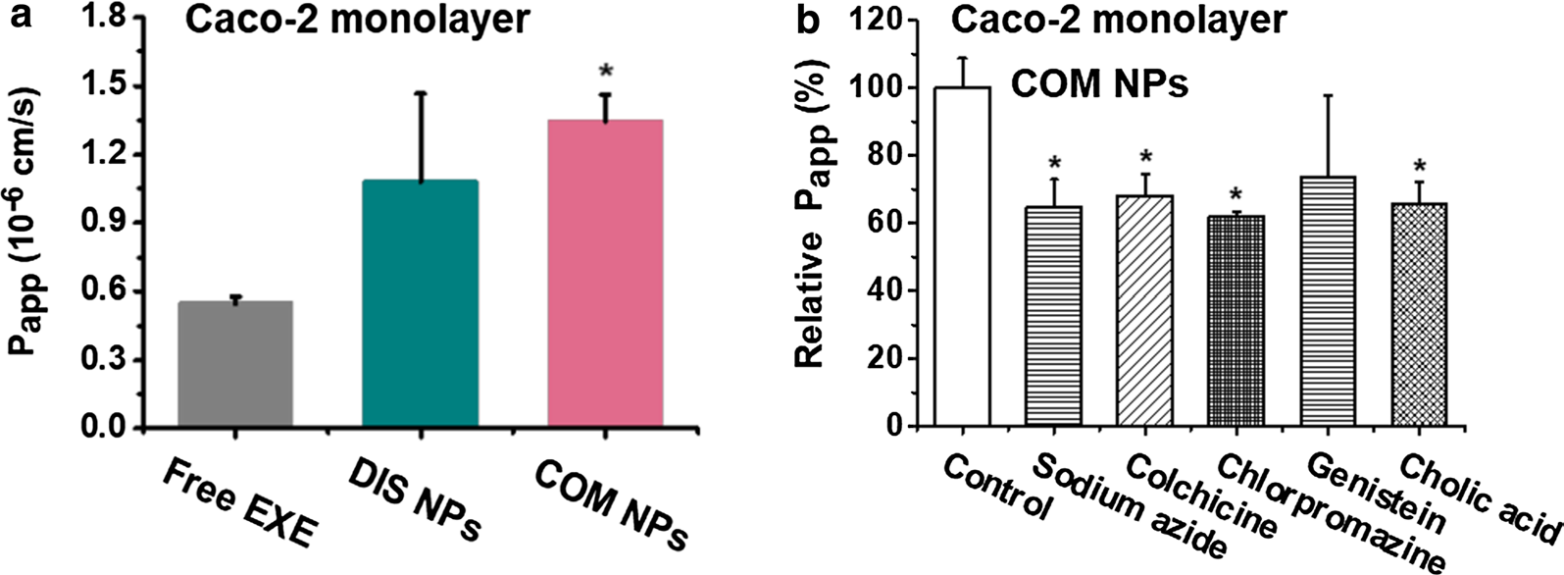
**a** P_app_ values of free EXE, DIS NPs and COM NPs in Caco-2 cell monolayers, *P < 0.05 compared with free EXE group; **b** relative P_app_ values of the COM NPs in Caco-2 cell monolayers treated with various inhibitors, *P < 0.05 compared with the COM NPs group without inhibitor (Control). (n = 3)

Caco-2 cell monolayer is a commonly used model to mimic the drug absorption in intestines [22, 47]. After 2 h incubation, the P_app_ values of DIS NPs and COM NPs were 2 and 2.4-fold of the P_app_ of free EXE, respectively (Fig. 2a). The transepithelial electrical resistance (TEER) result (Additional file 1: Fig. S4) revealed that the free EXE, DIS NPs and COM NPs had almost the same influence on the tight junctions of the cell monolayers. Therefore, the higher P_app_ values of the NPs were caused by the more NPs across the monolayers via transcellular transport rather than via paracellular transport.

The influences of various inhibitors on the transport of COM NPs across Caco-2 cell monolayers were investigated to understand the absorption mechanism. Caco-2 cells express ASBT and IBABP bile acid transporters [35]. Fig. 2b shows that free CA molecules caused a 35% reduction in the P_app_ of COM NPs, but the TEER values of the COM NPs groups with and without free CA molecules were almost the same as shown in Additional file 1: Fig. S4. This result demonstrated that part of the NPs transported the loaded EXE across the cell monolayers via bile acid transporters, and confirmed that the immobilized CA molecules at the NPs surfaces by hydrophobic interaction had transcytosis ability. Sodium azide reduced the P_app_ by 35%, suggesting that the transcytosis of the NPs was an energy-dependent process [48]. Colchicine, a micropinocytosis inhibitor, [49] reduced the P_app_ by 32%. Chlorpromazine, which can disrupt the assembly of clathrin, [50] reduced 38% of the P_app_. Genistein, an inhibitor of caveolae-mediated pathway, [51] did not significantly affect the transcytosis of the NPs. The results in Fig. 2b implied that COM NPs transported the loaded EXE through Caco-2 cell monolayers via bile acid transporters, micropinocytosis and clathrin-mediated transcellular pathways.

### In vitro permeability across excised small intestine walls of mice

**Fig. 3:**
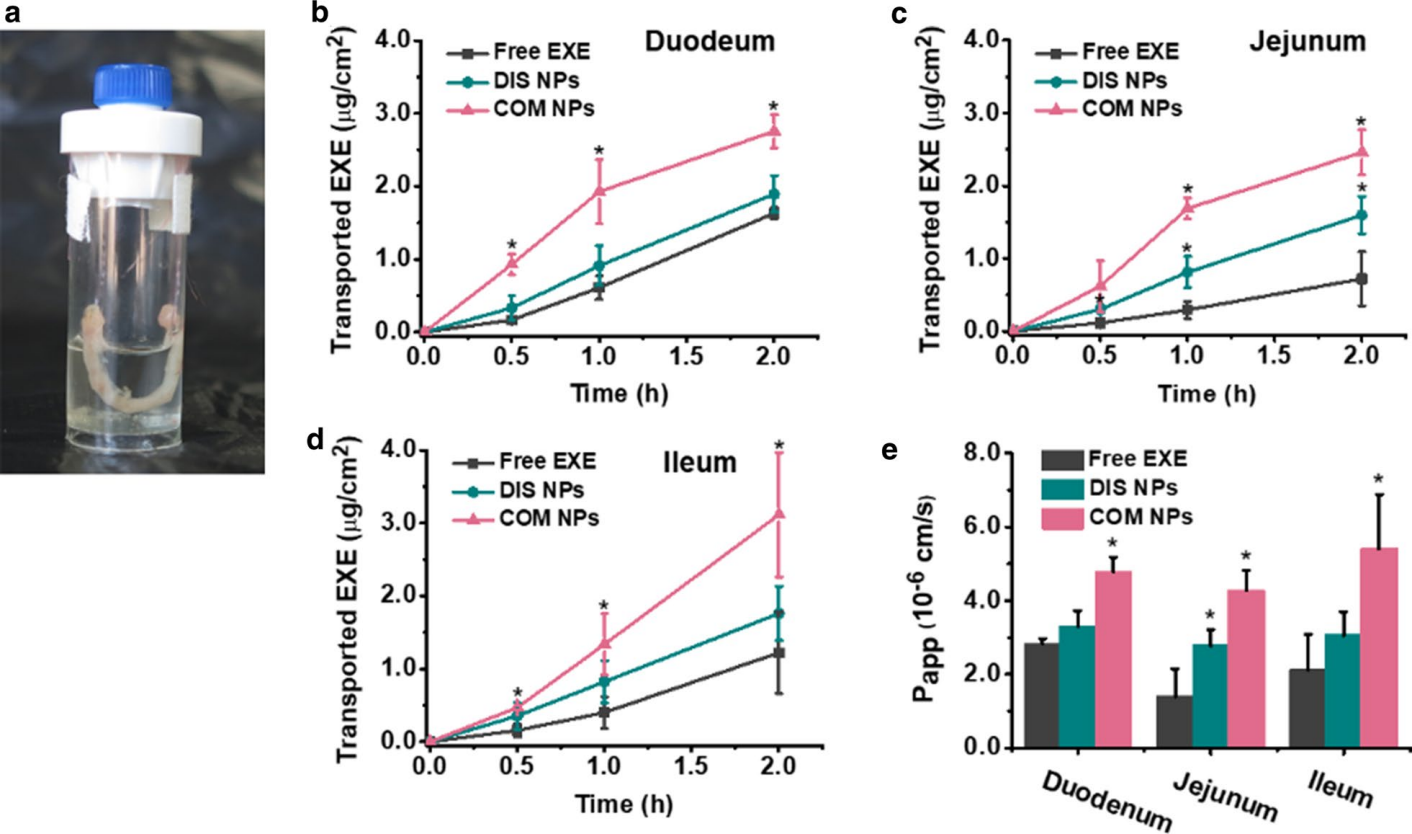
**a** Photo of the device for excised intestine permeability experiment. Cumulative EXE across **b** duodenum **c** jejunum and **d** ileum walls of the mice, and **e** P_app_ values of free EXE, DIS NPs and COM NPs after 2 h incubation. *P < 0.05 compared with free EXE group. (n = 3)

Both intestinal mucus and enterocyte affect the permeability of macromolecular drug. [52] To better mimic the in vivo absorption, the permeability abilities of free EXE, DIS NPs and COM NPs across excised duodenum, jejunum and ileum walls of mice were evaluated. As shown in Fig. 3, the EXE transport rates were in the order of COM NPs > DIS NPs > Free EXE. After 2 h incubation, the P_app_ values of the DIS NPs and COM NPs were 1.5-1.8-fold and 1.7-3.1-fold of the P_app_ values of the free EXE, respectively. These results indicate that COM NPs are superior to free EXE and DIS NPs in intestinal absorption.

### Gastrointestinal distribution of the EXE after oral administration

**Fig. 4:**
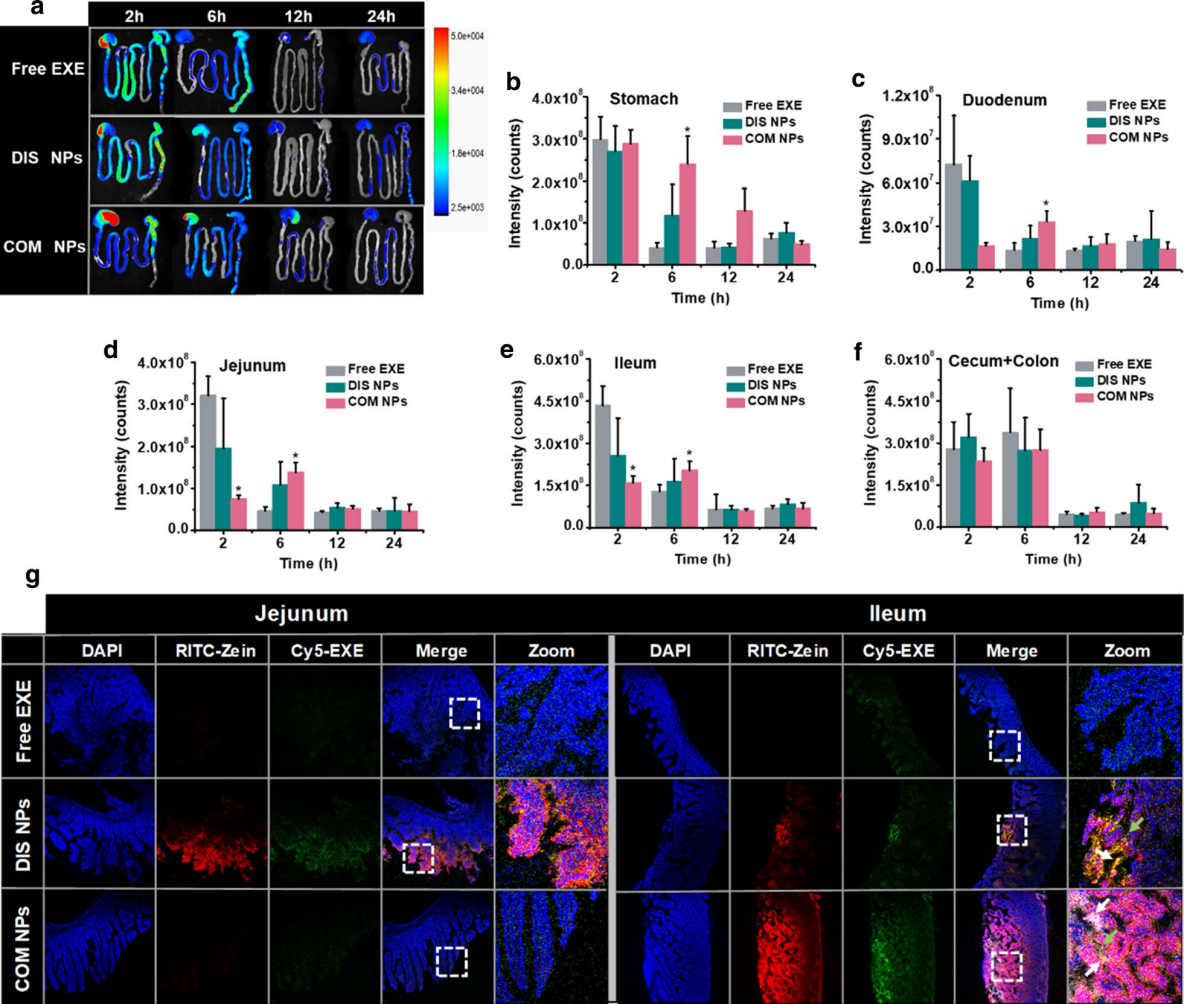
**a** Representative fluorescence images of the gastrointestinal tracts of the mice excised at 2, 6, 12 and 24 h after oral administrations with Cy5-labeled free EXE, DIS NPs and COM NPs; fluorescence intensities of **b** stomach, **c** duodenum, **d** jejunum, **e** ileum, and **f** cecum and colon of the mice after the oral administrations (n = 3), *P < 0.05 compared with free EXE group. **g** CLSM images of the jejunum and ileum sections of the mice excised after 2 h oral administrations with fluorescence-labeled free EXE, DIS NPs and COM NPs; the cell nuclei were stained with DAPI

Figure 4a–f show the fluorescent images and intensities of the gastrointestinal tracts of the mice after oral administrations with Cy5-labeled free EXE and NPs. The stronger fluorescent intensities at 6 and 12 h post-administration implied that the retention time of COM NPs was much longer than the time of free EXE and DIS NPs in stomach. For each group, the fluorescent intensity of the duodenum was weaker than the intensities of the jejunum and ileum at each time interval, indicating that the samples and/or degradation products stayed in jejunum and ileum for longer time than in duodenum. The fluorescent intensities implied that the COM NPs group had a slower EXE elimination rate in gastrointestinal tracts than the other groups.

### Distribution of the NPs in intestinal sections after oral administration

Cy5-EXE and RITC-Zein were used to prepare double fluorescent probes-labelled DIS NPs and COM NPs. Figure 4g shows the CLSM images of the jejunum and ileum sections of the mice after 2 h oral administrations with free Cy5-EXE and the NPs. In the duodenum images (not shown), all the groups did not show any detectable Cy5-EXE signal. In the jejunum and ileum images, free EXE group presented very weak Cy5-EXE signal. These results confirmed that most of the free EXE molecules could not get close to the intestinal villi and could not be absorbed by intestinal epithelial cells as reported in the literature [53]. For the two NPs groups, the fluorescence signals of Cy5-EXE and RITC-Zein were partially overlapped (yellow and white sites indicated by white arrows), indicating that a part of the DIS NPs and COM NPs did not dissociate during the digestion and absorption process. The total fluorescence intensities of the EXE and zein in COM NPs images were much stronger than the intensities in DIS NPs images, which was consistent with the result obtained by in vitro digestion that COM NPs had better EXE protection effect against protease digestion. Individual Cy5-EXE fluorescence signals were also detectable in the two NPs images (green sites indicated by green arrows), indicating that some of the EXE/CA/PC Complex and/or free EXE were released during the digestion and absorption process. The CLSM images revealed that DIS NPs were absorbed in jejunum and ileum, and COM NPs were mainly absorbed in ileum with much better absorption efficiency.

### EXE bioavailability in healthy mice and hypoglycemic efficacy in db/db mice after single oral administration

**Fig. 5:**
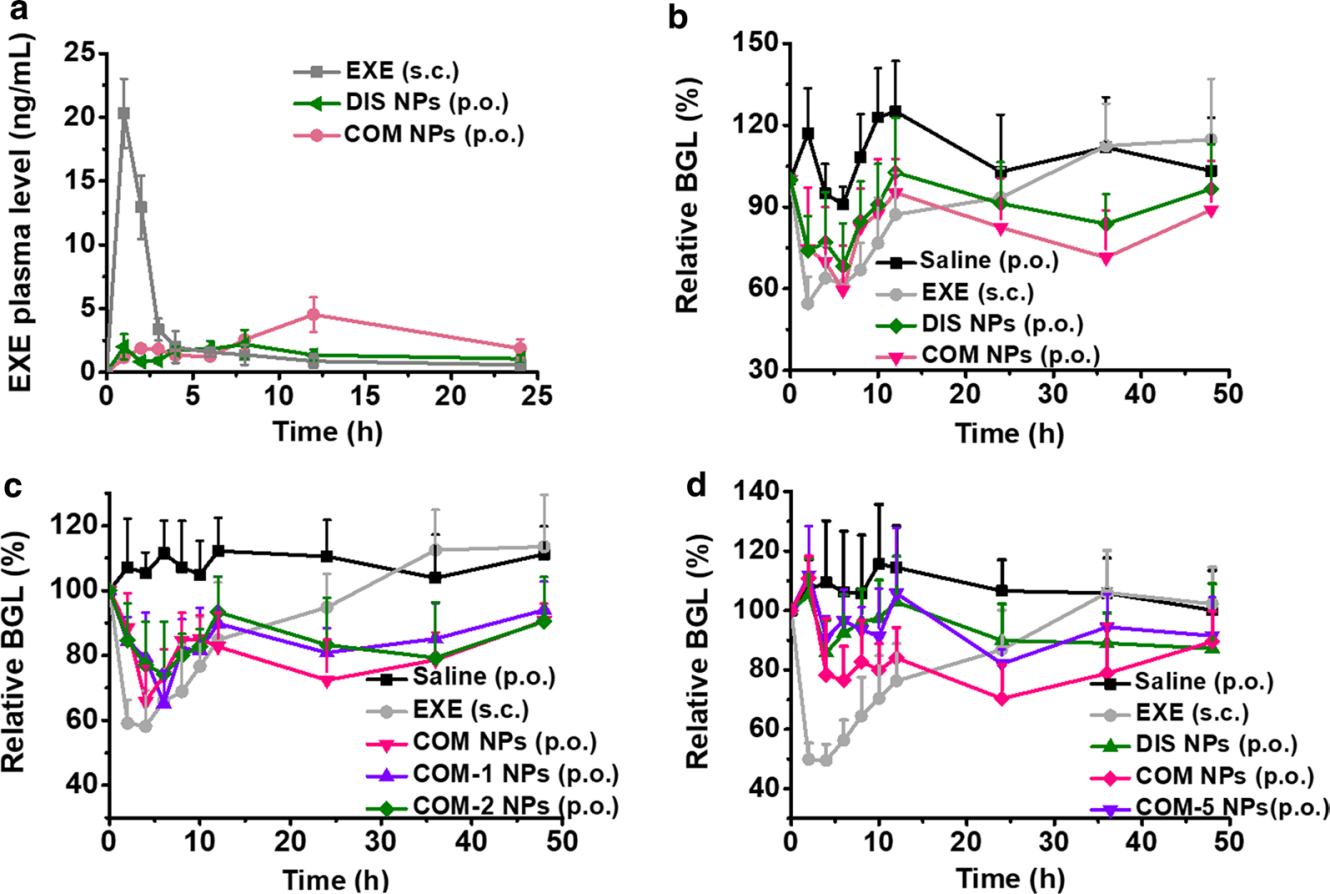
**a** EXE concentration changes in the plasma samples of the healthy mice (n = 3) after administrations with various formulations. **b**–**d** Relative BGL changes of the db/db mice (n = 6) after administrations with various formulations. The oral EXE dose was 0.6 mg/kg and the injected EXE dose was 0.06 mg/kg in **a**–**c**; the oral EXE dose was 0.78 mg/kg and the injected EXE dose was 0.078 mg/kg in **d**

Figure 5a shows the time-dependent EXE plasma concentration curves after oral administrations with the NPs and subcutaneous injection with free EXE solution. In clinical use, subcutaneous EXE injections have demonstrated glucoregulatory effect with sustained EXE plasma concentrations of 0.2–0.4 ng/mL [54, 55]. As shown in Fig. 5a, at 24 h post-administration, the EXE plasm concentrations of the COM NPs and DIS NPs oral groups were 1.88 ± 0.74 and 1.07 ± 0.33 ng/mL, respectively, significantly higher than the effective EXE plasma concentrations reported. Table 3 shows the corresponding pharmacokinetics data. The relative EXE oral bioavailabilities of the DIS NPs and COM NPs groups were 5.6% and 10.9%, respectively, verifying that COM NPs had higher EXE oral bioavailability than DIS NPs.

**Table 3:** Pharmacokinetics results after administrations with various formulations (n = 3)

Formulation	Admin route	Dose (mg/kg)	C_max_ (ng/mL)	T_max_ (h)	AUC_0–24_ (ng h/mL)	BA (%)
Free EXE	s.c.	0.06	20.3	1	59.2 ± 10.9	100
DIS NPs	p.o.	0.6	2.19	8	33.2 ± 2.8	5.6 ± 0.5
COM NPs	p.o.	0.6	4.52	12	64.4 ± 15.2	10.9 ± 2.6*

*P < 0.05 compared with the DIS NPs group

Figure 5b shows the relative BGL changes of the db/db mice after subcutaneous injection with free EXE solution and oral administrations with the NPs. Table 4 (Batch 1) shows the corresponding pharmacodynamics data. The relative BGL values of the saline group fluctuated between 90% and 120% of the initial value due to the free ingestions of water and standard chow. The appearance time (T_max_) of the maximal EXE plasm concentration (C_max_) shown in Fig. 5a was not consistent with the appearance time (T_min_) of the minimal BGL (BGL_min_) shown in Fig. 5b, which was probably caused by the free ingestion of food. The DIS NPs group presented similar BGL change trend but was less effective compared with the COM NPs group. The EXE oral pharmacological availabilities of DIS NPs and COM NPs were 13.1% and 18.6%, respectively, demonstrating that both NPs effectively improved the oral hypoglycemic efficacy of EXE and COM NPs had better efficacy. We further evaluated the oral hypoglycemic efficacies of several other formulations in Table 2. Figure 5c and Table 4 (Batch 2) show that COM NPs, COM-1 NPs and COM-2 NPs which contained 2, 0 and 4 mg/mL casein, respectively, did not show significant difference in the oral hypoglycemic efficacy. For COM NPs and COM-5 NPs, which contained 0.5 and 0 mg/mL CA, their EXE oral pharmacological availabilities in db/db mice were 12.8% and 6.7%, respectively, as shown in Fig. 5d and Table 4 (Batch 3). This result verified that the CA in COM NPs played a key role for the enhancement of the oral hypoglycemic efficacy. Table 4 (Batch 4) also shows that the formulations containing CA (COM NPs) and glycocholic acid (COM-8 NPs) had similar oral hypoglycemic efficacy.

**Table 4:** Pharmacodynamics results of the db/db mice after administrations with various formulations (n = 6)

Experiment batch	Formulation	Admin route	EXE dose (mg/kg)	BGL_min_ (%)	T_min_ (h)	T_diff_^a^ (h)	AAC_0–48_ (h %)	PA (%)
Batch 1	Free EXE	s.c.	0.06	54.5	2	2–12	724 ± 381	100
	DIS NPs	p.o.	0.6	68.3	6	2–10, 36	951 ± 404	13.1 ± 5.6
	COM NPs	p.o.	0.6	59.5	6	2–12, 36	1345 ± 701	18.6 ± 9.7
Batch 2	Free EXE	s.c.	0.06	58.1	4	2–24	677 ± 369	100
	COM NPs	p.o.	0.6	65.8	4	2–48	1366 ± 298	20.2 ± 4.4
	COM-1 NPs	p.o.	0.6	65.1	6	2–48	1127 ± 381	16.6 ± 5.6
	COM-2 NPs	p.o.	0.6	74.3	6	2–48	1153 ± 569	17.0 ± 8.4
Batch 3	Free EXE	s.c.	0.078	49.5	4	2–24	989 ± 452	100
	DIS NPs	p.o.	0.78	85.8	4	4, 24–36	696 ± 385	7.0 ± 3.9*
	COM NPs	p.o.	0.78	70.3	24	4–36	1267 ± 334	12.8 ± 3.4
	COM-5 NPs	p.o.	0.78	82.0	24	10, 24	664 ± 342	6.7 ± 3.5*
Batch 4	COM NPs	p.o.	0.6	64.2	4	–	855 ± 268	–
	COM-8 NPs	p.o.	0.6	71.9	2	–	923 ± 368	–

* P < 0.05 compared with the COM NPs group in the same batch

^a^Time points at which the relative BGL of the treatment group was significantly lower than the BGL of saline group (P < 0.05)

### Hypoglycemic and β-cell repair effects in db/db mice after 7-week consecutive oral administration

**Fig. 6:**
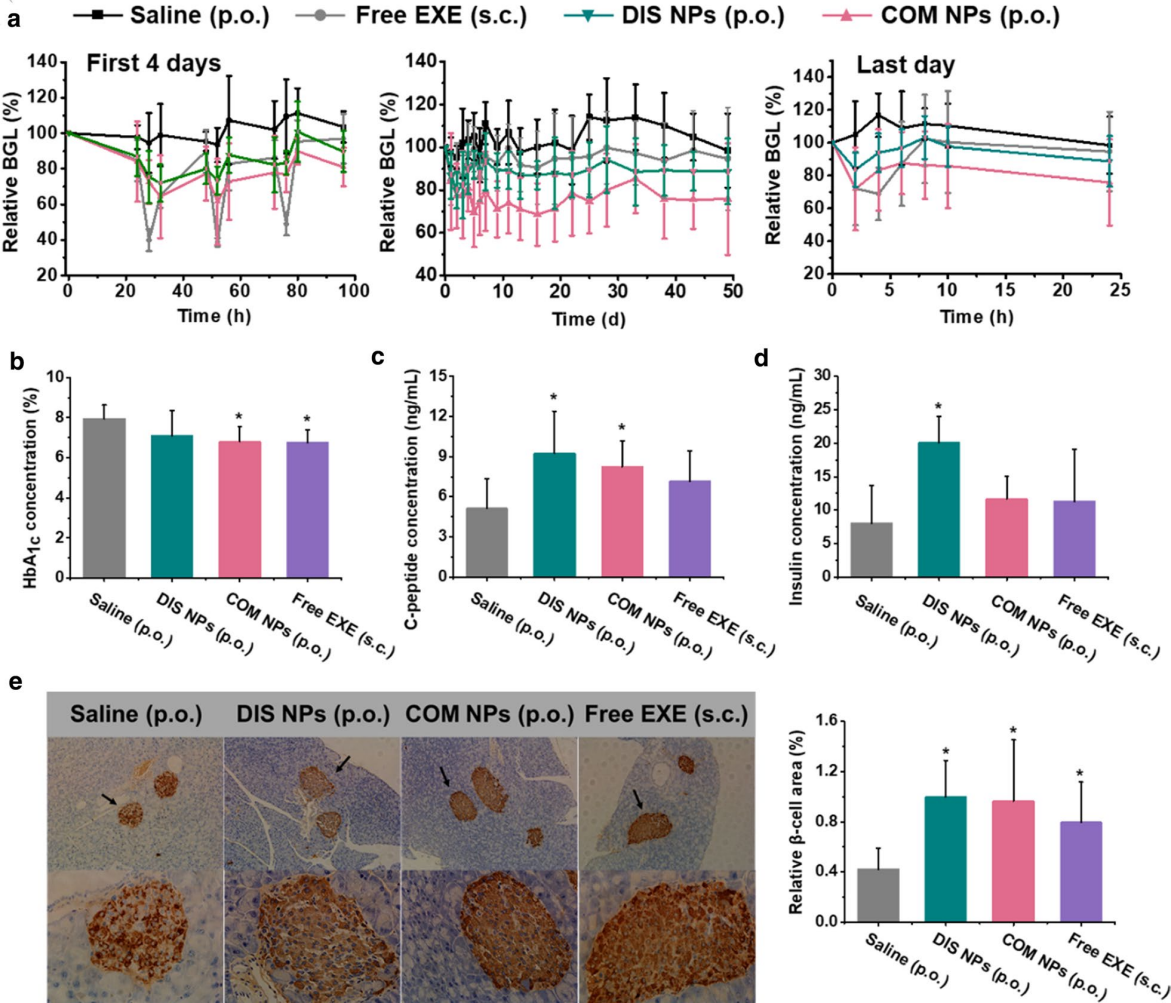
**a** Relative BGL changes of the db/db mice after oral administrations with DIS NPs and COM NPs at an EXE dose of 0.6 mg/kg and subcutaneous injection with free EXE solution at a dose of 0.06 mg/kg once daily for 7 weeks consecutively. **b** HbA_1c_, **c** C-peptide and **d** insulin concentrations of the db/db mice after the 7-week treatments. **e** Representative insulin-immunostained images and relative β-cell areas of the histological sections of the pancreatic islets from the db/db mice after the 7-week treatments. (n = 6), P < 0.05 compared with saline group

Long-term therapeutic effects in db/db mice were evaluated by 7 weeks of consecutive once daily administrations. The BGL of the free EXE injection group fluctuated sharply every day as shown in the BGL curve of the first 4 days and the curve of the last day (Fig. 6a). In contrast, the BGL fluctuations of the DIS NPs and COM NPs oral groups were much milder. After each oral administration, the hypoglycemic effect of the DIS NPs and COM NPs groups kept over 24 h. Compared with the saline group, the COM NPs oral group reduced the BGL by 18–36% while the DIS NPs oral group reduced the BGL by 10–25% during the experiment. The body weights of the three treatment groups were not significantly different compared with the weight of the saline group at each time point as shown in Additional file 1: Fig. S5.

After the 7-week treatments, several physiological indices of the db/db mice were measured. HbA_1c_ concentration in blood is an index for BGL control in the past several months [56]. Compared with the 7.95 ± 0.68% of the HbA_1c_ concentration of the saline group, the injection, COM NPs and DIS NPs groups decreased their HbA_1c_ concentrations to 6.74 ± 0.66% (P < 0.05), 6.79 ± 0.77% (P < 0.05), and 7.08 ± 1.29%, respectively (Fig. 6b). The HbA_1c_ concentrations demonstrated that the COM NPs oral group and injection group had comparable long-term hypoglycemic effect. As we know, the islet β-cells are progressively damaged in type 2 diabetics, and EXE is able to promote the β-cell repair and proliferation [6]. C-Peptide and insulin with a molar ratio of 1:1 are produced from insulin precursor secreted by β-cells. C-Peptide concentration in blood is an index to evaluate the insulin secretion function of β-cells because the insulin concentration is changeable due to the uptake and clearance by liver and periphery [57]. Figure 6c–e show the C-peptide and insulin concentrations in the plasma samples and the relative β-cell areas in the pancreatic islet sections of the db/db mice, respectively, after the 7-week treatments. Compared with the saline group, the DIS NPs oral group increased the C-peptide concentration by 81% and the β-cell area by 137% significantly, COM NPs oral group increased the C-peptide concentration by 61% and the β-cell area by 129% significantly, but the free EXE injection group only increased the β-cell area by 89% significantly. These results demonstrated that the DIS NPs and COM NPs oral groups had better β-cell repair effect than the injection group. According to the literature, [58, 59] oral administration of GLP-1 analog is more close to the physiological secretion of GLP-1 compared with subcutaneous injection. This factor may result in the better β-cell repair effect of the DIS NPs and COM NPs oral groups.

## Discussion

Both DIS NPs and COM NPs contained 6 ingredients. Zein acted as cement which bound the other ingredients in the NPs by hydrophobic interaction. Increasing the zein concentration resulted in higher EXE loading efficiency (Tables 1, 2). CA played a key role for the enhancement of the oral hypoglycemic efficacy as shown in Fig. 5d and Table 4 (Batch 3), and part of the NPs transported the loaded EXE across Caco-2 cell monolayers via bile acid transporters (Fig. 2b). Phospholipids are commonly used to produce inverted micelles [30]. We used PC to produce EXE/PC/CA complex micelles to increase the EXE loading efficiency in COM NPs. We also added free PC in DIS NPs to keep the same components and concentrations in DIS NPs and COM NPs. In vitro release and enzymatic degradation results (Fig. 1g, h) confirmed that HP, which is insoluble in acidic solution, prevented the release of the loaded EXE from the NPs and protected the EXE from digestion in simulated gastric fluid. Casein did not significantly increase the hyperglycemic efficacy of the NPs as shown in Fig. 5c and Table 4 (Batch 2). Casein acted as the cryoprotectant, therefore the NPs had excellent lyophilization, storage and re-dispersion stability. Because of the synergistic effects of the 6 ingredients, DIS NPs and COM NPs were effective oral delivery systems of EXE.

DIS NPs and COM NPs had the same components and concentrations, but COM NPs had higher EXE loading efficiency due to EXE/PC/CA complex micelles. It is reasonable that COM NPs had slower EXE release velocity, better EXE protection effect against digestion, longer retention time in gastrointestinal tract, better intestinal absorption, and there, higher EXE oral bioavailability and better oral hypoglycemic efficacy than DIS NPs. Unexpectedly, DIS NPs had comparable β-cell repair effect to COM NPs, including the substantial increases of insulin secretion by 81% and β-cell proliferation by 137%. Possibly, the different intestinal absorption site (Fig. 4g) and different distribution in the body resulted in the better β-cell repair effect of DIS NPs.

In both DIS NPs and COM NPs, CA molecules were embedded on the surfaces and in the interiors of the NPs by hydrophobic interaction. The embedded CA molecules enhanced the intestinal absorption and oral hypoglycemic efficacy of the NPs, whose function is similar to the functions of the CA and its derivative conjugated covalently to the surfaces of insulin-loaded nanoparticles and EXE-loaded liposomes [22, 36]. Obviously, most of the CA molecules were embedded in the interiors of the NPs. Zein was degradable in intestines by proteases, [44] the NPs were gradually eroded, and then the CA molecules in the interiors of the NPs were consecutively exposed to the surfaces to promote the absorption of the NPs and released EXE/PC/CA complex micelles. For the first time, this study proved that embedding absorption enhancer in zein nanoparticles by hydrophobic interaction is a practicable and effective strategy to improve the intestinal absorption and oral therapeutic effect of the loaded EXE. This strategy can be used for oral delivery of other peptide drugs.

## Conclusions

In this study, two types of hybrid zein nanoparticles containing EXE, cholic acid, phosphatidylcholine, hypromellose phthalate and casein were produced using anti-solvent precipitation method. Zein acted as the cement and the other ingredients were embedded in the nanoparticles by hydrophobic interaction. Casein acted as the stabilizer and cryoprotectant; the nanoparticles had excellent lyophilization, storage and re-dispersion stability. Hypromellose phthalate protected the loaded EXE from degradation in stomach. Cholic acid promoted the intestinal absorption of the loaded EXE via bile acid transporters. Due to the complex micelles of EXE, cholic acid and phosphatidylcholine, COM NPs had higher EXE loading efficiency, higher EXE oral bioavailability and better oral hypoglycemic efficacy in db/db mice than DIS NPs. COM NPs controlled the blood glucose level of the db/db mice well and the HbA_1c_ concentration significantly decreased to 6.79% during and after 7 weeks of consecutive once daily oral administration. Both orally administrated DIS NPs and COM NPs substantially improved the function and promoted the proliferation of the β-cells of the db/db mice after the 7-week administration. This study demonstrates that DIS NPs and COM NPs are promising systems for oral delivery of EXE, and using prolamin to produce multifunctional nanoparticles for oral delivery of peptide drug by hydrophobic interaction is a simple and effective strategy.

## Supplementary information

**Additional file 1:** Experiment methods: Lyophilization and storage of the NPs; in vitro EXE release; enzymatic degradation and HPLC analysis of EXE; cytotoxicity; in vivo biocompatibility; permeability of the EXE across Caco-2 cell monolayers; distribution of the EXE in gastrointestinal tract after oral administration. **Table S1.** Constituents and properties of various EXE-loaded nanoparticles without EXE/PC/CA Complex. **Fig. S1.** Photos of DIS NPs and COM NPs in pH 2.0 HCl solution and then in pH 7.4 PBS solution. **Fig. S2.** Caco-2 cell viabilities after 48 h incubations with DIS NPs and COM NPs at the NPs concentrations of 60-958 μg/mL (n = 3). **Fig. S3.** Representative hematoxylin–eosin stained histological images of the mouse organ sections excised after oral administrations with saline (control), DIS NPs and COM NPs at the NPs dose of 359 mg/kg (equal to 3 mg/kg EXE dose) once daily for 15 days consecutively. **Fig. S4.** Relative TEER changes of the Caco-2 cell monolayers after incubations with and then removes of FITC-labeled free EXE, DIS NPs and COM NPs as well as COM NPs plus free CA (n = 3). **Fig. S5.** Body weight changes of the db/db mice during the 7-week consecutive administration (n = 6).

## Data Availability

All data generated or analyzed during this study are included in this published article and its supplementary information file.
