# Supplementary material for: Oral delivery of exenatide-loaded hybrid zein nanoparticles for stable blood glucose control and β-cell repair of type 2 diabetes mice
Source: J Nanobiotechnology. 2020 Apr 28;18:67. doi: 10.1186/s12951-020-00619-0 (PMC7189518; doi:10.1186/s12951-020-00619-0)
Supplement: Supplementary file 1 — Additional file 1: Experiment methods: Lyophilization and storage of the NPs; in vitro EXE release; enzymatic degradation and HPLC analysis of EXE; cytotoxicity; in vivo biocompatibility; permeability of the EXE across Caco-2 cell monolayers; distribution of the EXE in gastrointestinal tract after oral administration. Table S1. Constituents and properties of various EXE-loaded nanoparticles without EXE/PC/CA Complex. Fig. S1. Photos of DIS NPs and COM NPs in pH 2.0 HCl solution and then in pH 7.4 PBS solution. Fig. S2. Caco-2 cell viabilities after 48 h incubations with DIS NPs and COM NPs at the NPs concentrations of 60-958 μg/mL (n = 3). Fig. S3. Representative hematoxylin–eosin stained histological images of the mouse organ sections excised after oral administrations with saline (control), DIS NPs and COM NPs at the NPs dose of 359 mg/kg (equal to 3 mg/kg EXE dose) once daily for 15 days consecutively. Fig. S4. Relative TEER changes of the Caco-2 cell monolayers after incubations with and then removes of FITC-labeled free EXE, DIS NPs and COM NPs as well as COM NPs plus free CA (n = 3). Fig. S5. Body weight changes of the db/db mice during the 7-week consecutive administration (n = 6). [file 12951_2020_619_MOESM1_ESM.docx]

**Additional file 1**

**Oral delivery of exenatide-loaded hybrid zein nanoparticles for stable blood glucose control and β-cell repair of type 2 diabetes mice**

Xiaoyan Bao^1^, Kang Qian^2^ and Ping Yao^1^*

^1^ State Key Laboratory of Molecular Engineering of Polymers, Collaborative Innovation Center of Polymers and Polymer Composite Materials, Department of Macromolecular Science, Fudan University, Shanghai 200438, China

^2^ Key Laboratory of Smart Drug Delivery, Ministry of Education, School of Pharmacy, Fudan University, Shanghai 201203, China

* Correspondence: [yaoping@fudan.edu.cn](mailto:yaoping@fudan.edu.cn).

**S1. Experiment methods**

**Lyophilization and storage stability of the NPs**

Freshly prepared FITC-COM NPs, FITC-COM-1 NPs (without casein, Table 2), and FITC-DIS NPs were lyophilized then re-dispersed in deionized water. The loading efficiencies of the re-dispersed NPs solutions were characterized. In addition, the lyophilized COM NPs and DIS NPs were stored at −20 °C, 2−8 °C and 25 °C for 6 months. At predetermined time intervals, the samples were re-dispersed in deionized water and the D_h_ values of the re-dispersed NPs were measured.

***In vitro* EXE release**

FITC-COM NPs and FITC-DIS NPs solutions of 0.5 mL were separately dialyzed (cutoff molecular weight 100 kDa, Spectra/Por Float-A-Lyzer G2, Spectrum Laboratories Inc.) against 5 mL pH 2.0 HCl solution at the first 2 h. After that, the release medium was changed to 5 mL PBS (10 mM phosphate buffer containing 0.15 M NaCl, pH 7.4). The release experiment was performed at 37 °C with 100 rpm shaking. At each predetermined time interval, 0.5 mL release medium was taken out and the same volume of fresh pre-warmed medium was supplemented. The FITC-EXE concentration in the release medium was analyzed on the fluorescence microplate reader.

**Enzymatic degradation and HPLC analysis of EXE**

Pepsin was dissolved in 0.1 M HCl solution with 8 mg/mL concentration, and pancreatin was dissolved in 10 mM pH 7.4 phosphate buffer with 8 mg/mL concentration. The NPs solution of 0.9 mL was mixed with 0.1 mL pepsin solution or pancreatin solution, and the mixed solution was incubated at 37 °C. At predetermined time interval, the sample was mixed with 1 mL ethanol to release the loaded EXE. After centrifugation at 10000 rpm for 4 min, 20 μL supernatant was immediately injected into a HPLC system (Agilent 1200, Agilent) equipped with a C18 column (Agilent Eclipse XDB-C18, 5 μm, 4.6 × 250 mm) for EXE quantitative analysis. The mobile phase was acetonitrile/water mixture containing 0.1 v% trifluoroacetic acid with linear gradient from A (30/70, v/v) to B (45/55) within 15 min, and the flow rate was 1.0 mL/min. The column temperature was 25 °C. The EXE absorbance was detected at 220 nm. Free EXE standard solution was analyzed at the same condition for EXE concentration calibration.

**Cytotoxicity**

Caco-2 cells were cultured in serum-containing medium (DMEM supplemented with 10 v% fetal bovine serum, 1 v% L-glutamine, 1 v% non-essential amino acids and 1 v% penicillin-streptomycin solution). The cells were seeded at a density of 5 × 10^3^ cells/well in 96 well plates and incubated at 37 °C for 24 h. After that, the medium was replaced by serum-free medium containing COM NPs or DIS NPs, and the cells were incubated at 37 °C for 48 h. Then, the medium was replaced by 120 μL MTS (3-(4,5-dimethylthiazol-2-yl)-5-(3-carboxymethoxyphenyl)-2-(4-sulfophenyl)-2H-tetrazolium) solution (16.7 v% in DMEM). After incubation at 37 °C in the dark for 2 h, the absorbance of the plates at 490 nm was measured on the fluorescence microplate reader.

***In vivo* biocompatibility**

Healthy male ICR mice were separately administrated orally with COM NPs and DIS NPs at a NPs dose of 359 mg/kg (equal to 3 mg/kg EXE dose) once daily. After 15 days of once daily administration consecutively, the mice were sacrificed, and the organs were surgically taken out. The hematoxylin-eosin stained organ sections were prepared as reported previously.[^1^](#_ENREF_1) The histological images of the sections were observed on the microscope.

**Permeability of the EXE across Caco-2 cell monolayers**

Caco-2 cells were seeded in 24-well transwell chambers and cultured for 14−21 days until the transepithelial electrical resistance (TEER) reached to 500 Ω·cm^2^ to obtain Caco-2 cell monolayers. After removing the culture medium, the apical side (AP) and basolateral side (BL) were washed with pre-warmed Hank's Balanced Salt Solution (HBSS) twice and then the cell monolayers were equilibrated in HBSS at 37 °C for 30 min. The HBSS was replaced with 0.2 mL sample in the AP and 0.6 mL fresh HBSS in the BL, and the cell monolayers were incubated at 37 °C. The sample was HBSS containing free FITC-EXE, FITC-DIS NPs or FITC-COM NPs with 50 μg/mL EXE concentration. At predetermined time intervals, 0.2 mL solution was collected from the BL, and the same volume of pre-warmed HBSS was supplemented. The FITC-EXE concentration in the BL solution was analyzed on the fluorescence microplate reader. The apparent EXE permeability (P_app_) was calculated using the following equation:

$$P_{app}=\frac{Q}{AC_{0}t}$$

where *Q* was the cumulative amount (ng) of the FITC-EXE transported into the BL, *A* was the area (0.33 cm^2^) of the Caco-2 cell monolayer, *C_0_* was the initial concentration (ng/mL) of the FITC-EXE in the AP, and *t* was the duration time (s) of the experiment.

The permeability experiments of FITC-COM NPs were also performed in the presence of various inhibitors to identify the transcellular mechanism. After the transwell was washed with pre-warmed HBSS, the cell monolayers were incubated in the inhibitor solution at 37 °C for 30 min. The inhibitor solution was HBSS containing 100 nM sodium azide, 10 μg/mL chlorpromazine, 4 μg/mL colchicine, 100 μg/mL genistein or 100 μM cholic acid. After that, the solution in the AP was removed, fresh inhibitor solution containing FITC-COM NPs with 50 μg/mL EXE concentration was added, and the cell monolayers were incubated at 37 °C for another 2 h. Then, the FITC-EXE concentration in the BL solution was analyzed and P_app_ was calculated as described above.

TEER values were measured to assess the impact of free EXE and the NPs on the tight junctions of the Caco-2 cell monolayers. The cell monolayers were equilibrated in HBSS for 30 min and the TEER values were measured using an electrical resistance system (ERS-2, Millipore). Subsequently, the HBSS was replaced with 0.2 mL HBSS containing free FITC-EXE, FITC-DIS NPs or FITC-COM NPs with 50 μg/mL EXE concentration in the AP and 0.6 mL fresh HBSS in the BL, and the TEER values were measured after 0, 1 and 2 h of the incubation. Then, the AP and BL were washed thrice with pre-warmed HBSS, the monolayers were incubated in fresh HBSS for another 2 h, and the TEER values were measured again. In addition, the TEER values of the monolayers in the presence of 100 μM cholic acid and FITC-COM NPs together were measured parallelly.

**Distribution of the EXE in gastrointestinal tract after oral administration**

Cy5-labelled EXE (Cy5-EXE) was synthesized according to the literature.[^2^](#_ENREF_2) Cy5-EXE was used to prepare Cy5-DIS NPs and Cy5-COM NPs. Healthy male ICR mice were fasting for 12 h with free access to water before the experiment. After the oral administration of Cy5-EXE, Cy5-DIS NPs or Cy5-COM NPs with 1.2 mg/kg EXE dose, the mice were sacrificed at 2, 6, 12 and 24 h post-administration. The gastrointestinal tracts were taken out and the external blood was washed away. *Ex vivo* fluorescence images of the gastrointestinal tracts were acquired on a small animal imaging system (In Vivo Xtreme, Bruker) and the fluorescence intensities of the gastrointestinal tracts were measured.

**S2. Results**

**Table S1**  Constituents and properties of various EXE-loaded nanoparticles without EXE/PC/CA Complex

| Formulation | EXE | Zein | PC | CA | HP | Casein | P188 | D_h_ (nm) | PDI | LE (%) |
| --- | --- | --- | --- | --- | --- | --- | --- | --- | --- | --- |
|  | mg/mL | | | | | | |  |  |  |
| DIS NPs | 0.08 | 5 | 1 | 0.5 | 1 | 2 | 0 | 177±1 | 0.06±0.04 | 53.6±0.3 |
| DIS-1 NPs | 0.08 | 3.75 | 1 | 0.5 | 1 | 2 | 0 | 187±2 | 0.16±0.02 | 44.9±1.8 |
| DIS-2 NPs | 0.08 | 2.5 | 1 | 0.5 | 1 | 2 | 0 | 168±1 | 0.13±0.03 | 31.1±1.9 |
| DIS-3 NPs | 0.0167 | 5 | 0 | 0.12 | 0.5 | 2 | 0 | 148±1 | 0.14±0.02 | 55.1 |
| DIS-4 NPs | 0.0167 | 3.75 | 0 | 0.12 | 0.5 | 2 | 0 | 121±2 | 0.16±0.02 | 46.4 |
| DIS-5 NPs | 0.0167 | 2.5 | 0 | 0.12 | 0.5 | 2 | 0 | 116±2 | 0.10±0.03 | 24.2 |
| DIS-6 NPs | 0.0167 | 5 | 0 | 0.12 | 0.5 | 2 | 4 | 153±1 | 0.09±0.01 | 66.2 |
| DIS-7 NPs | 0.0167 | 5 | 0 | 0.12 | 0.5 | 0 | 8 | 114±1 | 0.08±0.03 | 82.8 |
| DIS-8 NPs | 0.08 | 5 | 0 | 0.5 | 1 | 2 | 0 | 192±4 | 0.10±0.02 | 56.5 |
| DIS-9 NPs | 0.0167 | 2.5 | 1 | 0.12 | 1 | 2 | 0 | 128±1 | 0.16±0.01 | 32.1 |
| DIS-10 NPs | 0.16 | 5 | 1 | 0.5 | 1 | 2 | 0 | 168±1 | 0.15±0.03 | 60.7 |
| DIS-11 NPs | 0.5 | 5 | 1 | 0.5 | 1 | 2 | 0 | 160±1 | 0.12±0.05 | 62.1 |

**Fig. S1**  Photos of DIS NPs and COM NPs in pH 2.0 HCl solution and then in pH 7.4 PBS solution.

**Fig. S2** Caco-2 cell viabilities after 48 h incubations with DIS NPs and COM NPs at the NPs concentrations of 60−958 μg/mL (n = 3).


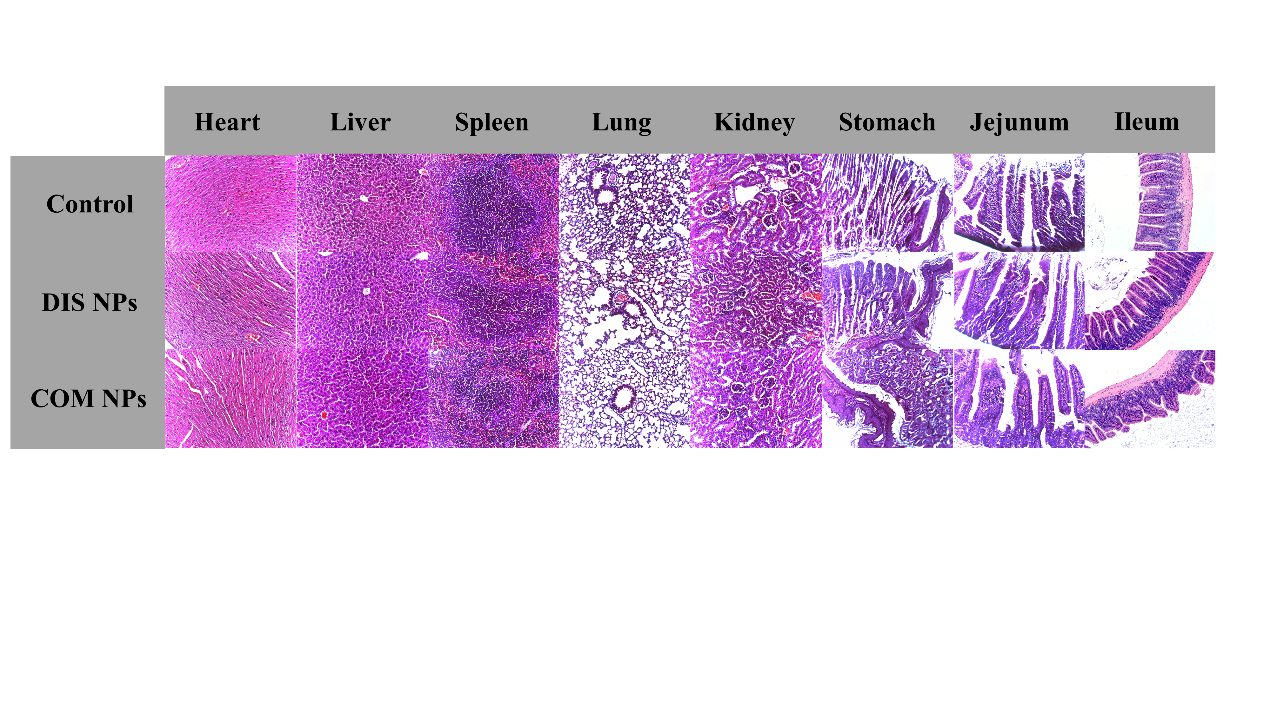


**Fig. S3** Representative hematoxylin-eosin stained histological images of the mouse organ sections excised after oral administrations with saline (control), DIS NPs and COM NPs at the NPs dose of 359 mg/kg (equal to 3 mg/kg EXE dose) once daily for 15 days consecutively.


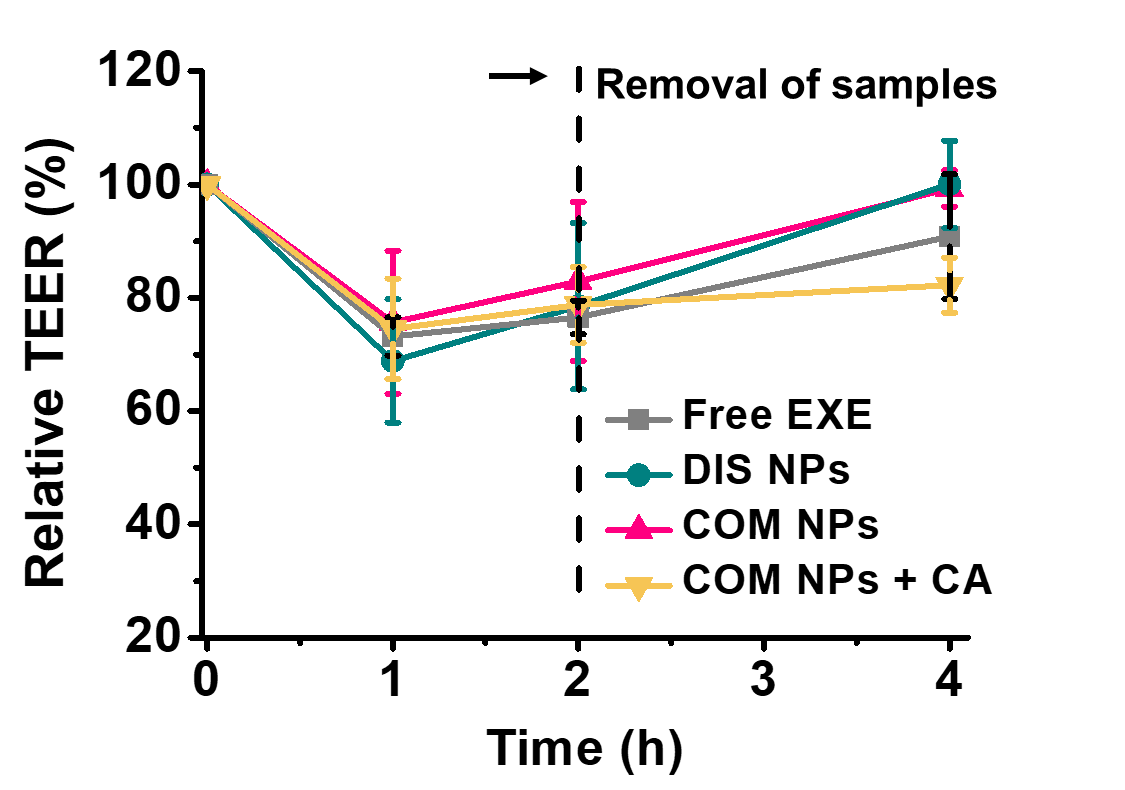


**Fig. S4**  Relative TEER changes of the Caco-2 cell monolayers after incubations with and then removes of FITC-labeled free EXE, DIS NPs and COM NPs as well as COM NPs plus free CA (n = 3).

**Fig. S5** Body weight changes of the db/db mice during the 7-week consecutive administration (n = 6).

**References**

1. Zhang Z, Cai H, Liu Z, Yao P. Effective Enhancement of Hypoglycemic Effect of Insulin by Liver-Targeted Nanoparticles Containing Cholic Acid-Modified Chitosan Derivative. Mol Pharm. 2016;13:2433-42.

2. Zhang Z, Li H, Xu G, Yao P. Liver-targeted delivery of insulin-loaded nanoparticles via enterohepatic circulation of bile acids. Drug Deliv. 2018;25:1224-33.
